# Understanding the workforce that supports Māori and Pacific peoples with type 2 diabetes to achieve better health outcomes

**DOI:** 10.1186/s12913-022-08057-4

**Published:** 2022-05-19

**Authors:** Tania Mullane, Matire Harwood, Isaac Warbrick, Taria Tane, Anneka Anderson

**Affiliations:** 1 Whitireia Institute, DX Box: SX33459, Porirua, Wellington New Zealand; 2 Department of General Practice and Primary Care, Faculty of Medical and Health Sciences, University of Auckland Private Bag 92019, Auckland Mail Centre, Auckland, 1142 New Zealand; 3 Faculty of Health and Environmental Sciences, Auckland University of Technology Private Bag 92006, Auckland, 1142 New Zealand; 4 Faculty of Medical and Health Sciences, Te Kupenga Hauora Māori, University of Auckland Private Bag 92019, Auckland Mail Centre, Auckland, 1142 New Zealand; 5 Faculty of Medical and Health Sciences, Te Kupenga Hauora Māori, University of Auckland Private Bag 92019, Auckland Mail Centre, Auckland, 1142 New Zealand

**Keywords:** Diabetes, Indigenous, Qualitative, Māori, Pacific, Workforce

## Abstract

**Background:**

Prevalence of Type 2 diabetes mellitus (T2DM) is high among Māori and other Pacific Island peoples in New Zealand. Current health services to address T2DM largely take place in primary healthcare settings and have, overall, failed to address the significant health inequities among Māori and Pacific people with T2DM.

Culturally comprehensive T2DM management programmes, aimed at addressing inequities in Māori or Pacific diabetes management and workforce development, are not extensively available in New Zealand. Deliberate strategies to improve cultural safety, such as educating health professionals and fostering culturally safe practices must be priority when funding health services that deliver T2DM prevention programmes.

There is a significant workforce of community-based, non-clinical workers in South Auckland delivering diabetes self-management education to Māori and Pacific peoples. There is little information on the perspectives, challenges, effectiveness, and success of dietitians, community health workers and kai manaaki (KM) in delivering these services.

This study aimed to understand perspectives and characteristics of KM and other community-based, non-clinical health workers, with a focus on how they supported Māori and Pacific Peoples living with T2DM to achieve better outcomes.

**Methods:**

This qualitative study undertaken was underpinned by the Tangata Hourua research framework. Focus groups with dietitians, community health workers (CHWs) and KM took place in South Auckland, New Zealand. Thematic analysis of the transcripts was used to identify important key themes.

**Results:**

Analysis of focus group meetings identified three main themes common across the groups: whakawhanaungatanga (actively building relationships), cultural safety (mana enhancing) and cultural alignment to role, with a further two themes identified only by the KM and CHWs, who both strongly associated a multidisciplinary approach to experiences of feeling un/valued in their roles, when compared with dietitians. Generally, all three groups agreed that their roles required good relationships with the people they were working with and an understanding of the contexts in which Māori and Pacific Peoples with T2DM lived.

**Conclusions:**

Supporting community based, non-clinical workers to build meaningful and culturally safe relationships with Māori and Pacific people has potential to improve diabetes outcomes.

## Background

Currently, 8% of the world’s population have type 2 diabetes mellitus (T2DM) [1] with significant increases expected in developing countries, [2–5] and Indigenous people’s populations due to a move away from health eating, active lifestyles, and controlled urbanization, [6]. Additionally Indigenous peoples have higher rates of diabetes complications such as eye, kidney, vascular disease and significantly advanced rate of mortality compared to non-Indigenous people [7] suggesting inequities in diabetes management to reduce the risk for such complications. Such is the case in in Aotearoa New Zealand (hereafter referred to as NZ) with Māori, the Indigenous people of NZ, and Pacific Peoples experiencing significantly higher rates for diabetes and its complications with increased exposure to the wider determinants for diabetes and reduced access to treatments [8].

Community-centred care plays an important role in addressing these inequities through the provision of lifestyle advice, diabetes and self-management education, and medication [9]. In NZ a range of workers, including general and nurse practitioners, nurses, community health workers and dietitians, provide these services. Further efforts to improve access to Māori and Pacific Peoples have tended to focus on outreach by conventional services such as nurse clinics [10] and dietitians [11]. However, these may not be effective in achieving equitable outcomes for Māori and Pacific Peoples with T2DM. Further, there is little information about what kind of workforce, or workforce development, is required to improve diabetes outcomes for Māori and Pacific Peoples.

In 2016 the National Hauora Coalition (NHC), a Māori-led primary health organisation, received funding to test Mana Tū, an innovative programme for Māori and Pacific Peoples with T2DM. Mana Tū (which means ‘to stand with authority’), a whānau ora approach to long-term conditions, is a Māori-led, Kaupapa Māori approach to supporting people living with poorly controlled T2DM, and their wider whānau (family members) [12]. Kai manaaki (KM) are skilled case managers who are embedded within a primary care service as core workforce; their case management approach supports diabetes self-management with Māori and Pacific Peoples living with poorly controlled T2DM [13].

In this paper we present a study that aimed to better understand the KM workforce compared with other diabetes workers. The main objective of this research was to investigate and compare the experiences for the main groups of community-based, non-clinical workers currently supporting Māori and Pacific Peoples and their whānau (families) with diabetes in South Auckland NZ – KM, Community Health Workers and Dietitians. The ultimate objective is to use the findings to inform future strategies for Māori and Pacific health workforce development, to improve the delivery of services and diabetes outcomes for Māori and Pacific Peoples.

## Methods

A qualitative study was undertaken within the Tangata Hourua research framework, which was developed by members of this research team, with the aspiration to contribute positively to Māori and Pacific knowledge and wellbeing [14]. The Tangata Hourua aims to uphold the rights of Indigenous peoples to have their knowledge and culture embedded in research by drawing upon Kaupapa Māori and pan-Pacific values and methodologies. In addition, the Tangata Hourua framework addresses the growing trend of Māori and Pacific people to be researched together, increasing numbers of those who identify as both Māori and Pacific and lack of frameworks that culturally address and reflect these approaches and populations [14].

Specifically, the study aimed to gain an understanding of the experiences for the main groups of community-based, non-clinical workers currently supporting Māori and Pacific Peoples and their whānau (families), rather than a broad representation of all health workers who deliver T2DM programmes. Focus groups were determined to be the most appropriate method to obtain this information, presenting an excellent opportunity to focus on a particular issue, privileging the subjective knowledge and life experiences of respondents [15]. Further, focus group methods align with important Māori and Pacific principles including mana-enhancement (acknowledging the contribution and expertise of each participant), mahitahi (co-operation between participants and researchers) and manaakitanga (reciprocity) by providing a collective forum for participants to share their own experiences and contribute to improving the services that they know intimately [16].

The focus groups were conducted with the three main workforce groups working in South Auckland, New Zealand. We chose this region for its ethnic profile, as 13% of the population identify as Māori, representing 24% of all Māori in New Zealand [17], and 64% of New Zealand’s Pacific population currently lives in Auckland, representing 15% of the Auckland population [17]. The three main workforces, KM, community health workers (CHWs) and dietitians, participated in three separate focus groups. Potential participants were recruited by sending an invitation to organisations employing dietitians, CHWs and KM. Once we had received more than six responses from one of the three workforces, we arranged focus group discussions which lasted 60–70 minutes and took place at work sites and at times that suited participants. Food and refreshments were provided for each group discussion. Informed consent and confidentiality agreements were obtained from all participants at the start of each meeting, and demographic information was collected (see Table 1).

**Table 1:** Profile of Focus Groups

Focus Group	Number	Demographic Profile of the Focus Groups	
		Ethnicity – self- identified	Gender
Kai Manaaki	Seven	Six Māori One Indo-Fijian	All female
Community Health Workers	Eight	Two Māori Two Pacific Peoples (Cook Island Māori and Samoan) Four New Zealand European	All female
Dieticians	Seven	Three Pacific Peoples (Tongan and Cook Island Māori) Four New Zealand European	All female

The guiding questions were:

1. What can you tell me about your role?
2. What do you think is important to consider in working with Māori and Pacific Peoples living with T2DM?
3. How do you know if you are effective?

The primary researcher transcribed the recorded focus group discussions verbatim. Themes arising from the data were analysed and informed by the Tangata Hourua model’s core Māori and Pacific values and concepts as a pou (pillar) to guide data analysis and theming. This is to ensure the authentic voices of Māori as Indigenous to New Zealand and the Pacific Peoples of Te Moana Nui a Kiwa (the Pacific Ocean) can be heard.

## Results

Table 1 presents the number and demographic profile of participants for each focus group. The focus groups reflected the fact that women make up the great majority of the New Zealand health care workforce [18], with all participants being female. Analysis of the data identified common themes: whakawhanaungatanga (actively building relationships), cultural safety and cultural alignment. However, there were also two key differences for KM and CHWs who (1) preferred a multi-disciplinary team (MDT) approach, and (2) described their experiences of feeling un/valued in their roles, when compared with dietitians. The themes are presented here.

### Key data themes

#### Culturally safe practice

As one worker summarised, cultural safety in practice meant using authentic language, sharing decision-making and genuinely wanting the best for clients:

“I find the language that you use has to be simple … non-judgmental lens, taking that judgement out, because they are already feeling so crap … we don't use jargon from the clinic we use everyday language that we/they know. It’s about creating a supportive environment for people to thrive in and that’s also very important” – KM, Māori.

Culturally safe practice required time. The concept of ‘time for equity’ allowed appointments to go as long as necessary to “show you value them and their time” when we “just chill out listen, then they talk”. Consequently, clients told workers that they felt heard, and were more likely to “actually, come up with the solution. You [health worker] don’t have to do anything, don’t make assumptions that we know any more than they do they know” – KM, Māori.

Going further, time created the opportunity to shift power from the health worker to the client, as one KM acknowledged “sometimes our appointments would go for an hour or so, ‘cause they just needed that time to sort of warm up and then really really get out what they were trying to say or wanted to know … there’s a valuing of their time and what they’re talking about … it’s a two-way thing it’s not that anyone is better but because we had the time just sit there and listen” – KM, Māori.

Cultural concordance – knowing the context within which Māori and Pacific Peoples with T2DM lived – was associated with better engagement and, subsequently, better outcomes. As one worker said, “these are my brothers and sisters. I know what works and what doesn’t.” Pacific workers were appreciated by the non-Pacific workers because:

“Being in the community and working with Pacific Peoples means language is important, so having a Pacific CHW makes such a difference with supporting us with language, but also with the engagement, she can engage with them in a way that I can’t and that’s good.”

However, there were major barriers to culturally safe practice. For example, there were inadequate culturally specific resources to work with: “trying to find pictures [of healthy food options] that represent Māori or Pasifika people is really hard sometimes” with dietitians commenting that most resources were “very white” (CHW, NZ European). This limited their ability to be culturally safe.

Another barrier was the lack of Māori and Pacific workers in the diabetes sector. Living in Māori and Pacific communities, Māori and Pacific workers were more likely to be “Understanding [of] everybody’s work timetable, kids’ timetable, how are we going to put your food and medication around what you are already doing, and how can we adjust that for your whole family’s benefit. Go out to [local food] markets and understand where they shop and what food they eat” – CHW, NZ European.

All agreed that building Māori and Pacific workforce capacity in T2DM management was important in order to reflect the population with the highest incidence of T2DM. The dieticians, especially, strongly advocated that any workforce development should attract and retain more Māori and Pacific into health professional roles, which also supported the District Health Board goals of increasing more Māori and Pacific staff.

A key barrier was building cultural safety practice in the non- Māori, non-Pacific workforce. A Pākehā (European) worker described Pākehā feeling overwhelmed and almost paralysed in their practice with Māori and Pacific peoples, and seemed to direct the fault at clients who were less likely to listen or trust non-Māori, non-Pacific workers:

“I think potentially as a non-Māori and non-Pacific there’s a barrier there right at the start in terms of (from client/whanau perspective) ‘am I going to listen, am I going to understand the … mistrust with what’s happened before’ so how can we start to engage them?” – Dietician, NZ European.

There was also reflection by these workers that their professional views on the importance of a cultural approach, and it being essential to integrate into practice to build genuine relationships and achieve better diabetes outcomes for clients and their whānau, had changed. With time and experience their ideas on what was good or bad practice had developed:

“quite a few years ago I would have thought that was like such bad practice [to spend time developing meaningful relationships rather than take a clinical history]. Now I think it's so important, because I don't need a diet history to be able to do my job and do it well. So yeah, just massive changes of thought and process around how you do things and then obviously that's that for another time at building relationships … sometimes I'll spend an entire session just chatting [in order to know each other].

Such reflections by the European practitioners in our study suggest that there is hope in terms of the ability to transform practice from not only an individual but collective perspective. Every one of the New Zealand European dieticians spoke about their self-awareness of unconscious biases, with one dietician acknowledging that some clients did not want to work with them because they were Pākehā (New Zealand European), which forced them to reflect on awareness and responsibility of cultural safety. Importantly they acknowledged that when they did not practice in a culturally safe or appropriate way, they did not fulfil their job or provide an effective service.

#### Whakawhanaungatanga

Whakawhanaungatanga (noun), an important cultural concept for Māori and Pacific peoples, emerged in the focus group discussions. It is formally defined as the “process of establishing relationships, relating well to others” [19]. For the focus group participants, it occurred between provider and clients, but required “treating my patients like they are my sister, my brother, my whānau, which quickly establishes a relationship.”

Despite the word whānau (family) appearing in whakawhanaungatanga, it is more than just making family links – it supports people to establish and maintain personal and culturally relevant connections [20]. KM in particular were most aware of its significance, with one stating “Talking [with clients] about their families and about them first before diving into medical stuff … build up a relationship, establishing a really good rapport, it’s connection. …” As described here, it should happen early in the consultation and well before more traditional questioning/informing.

As a Pacific dietician acknowledged, whakawhanautanga came easily to the Māori and Pacific staff as it learnt over their lifetime, and reflects their collective cultural values and worldviews:

“We [Pacific Peoples] are more like a group rather than self … [in] understanding them and also understand how the Pacific and Māori live and what mattered to them, fitting into their world view” – Dietitian, Pacific.

As stated in one focus group “Trust building is important and how we build trust to them and then they tell things to us; which allows clients to feel safe.” Whakawhanaungatanga provided a framework in which trust was built. As a result, there was better rapport, and the focus group participants said that clients were more open to taking on advice or interventions, resulting in better outcomes down the track.

For non- Māori, non-Pacific health workers “Things like whakawhanaungatanga is vital, nobody ever writes about it, [yet] it’s intrinsic to this community” –CHW, NZ European.

They felt strongly that more could be done with their non-Māori, non-Pacific colleagues to highlight the importance of whakawhanaungatanga when working with Māori and Pacific Peoples and communities; and that it should be a core competency for all the participants’ professional roles. Resources to guide its use and inform users on the quality of their whakawhanaungatanga in practice, were sought.

#### Culturally safe spaces

Delivering services in traditional cultural settings such as marae (traditional meeting houses) for Māori and church for Pacific Peoples were effective for shifting power to clients, due to their experience of cultural affirmation. One CHW described the importance of traditional cultural settings or principles for services for Māori and Pacific Peoples:

“Being based on the whenua [land] of a marae is key to what we do and how we do it. I don’t think we can say that any other clinic in the whole of Auckland has as higher Māori ethnicity as we do. [It’s a space] where they can just be and interact with others and be heard” – CHW, Māori.

Participants spoke of their own enjoyment when “being on the marae and having the marae support, working within an organisation that is guided by kaupapa Māori values … it’s a privilege” – KM, Māori.

Importantly, ‘the [marae-based] clinic works like a centre of the community, so people feel like they can pop in anytime, walk in clinics to see someone; There is also a spiritual component … the idea of community, the idea of inclusion” – CHW, Māori. It was considered an example of best practice, and other providers looked to them as a role model and a centre of excellence.

### Multidisciplinary team approach

The KM and CHWs both worked as part of a multidisciplinary teams (MDTs) and felt that this was best for managing T2DM for Māori and Pacific Peoples. An MDT approach was strength-based in the sense that different members of the health team had different roles based on their strengths or expertise. They described the importance of working in partnership with non-health service providers, including social or housing services, which facilitated access to interventions that addressed the wider determinants for health, such as poverty. For example, one KM described supporting a client to study for and sit their driver’s licence, which then enabled them to attend job interviews and finally attain employment.

The KM in particular felt that being part of a wider team meant that they contributed to the wider environment, and this had an impact on the health and wellbeing of the whole community. This influence occurred at multiple levels. For example, in the primary care clinics, they participated in team meetings, shared experiences and learnings about good practice, and advocated for clients and their whānau. One example was teaching clinicians (GPs and nurses) how they could do more to remove barriers to diabetes care. They knew where to locate or refer people for services and dismissed siloed care:

“I don’t know anyone outside the marae because we are so used to using who [multiple health and social service workers] we have here” – KM, Māori.

The KM and CHW spoke in depth about the importance of a multi-disciplinary approach to their role. In contrast a multidisciplinary approach was not mentioned by the Dieticians, this could be interpreted in several way, one of which is that they did not see it as important, but rather saw themselves in more of an independent practitioner role due to their status as a ‘regulated health role’ as opposed to the KM and CHW who are regarded as ‘non-regulated health roles’. The ethnic makeup of each of the group may have bearing on what was discussed.

### Feeling un/valued

CHWs described feeling marginalised by some diabetes services because they were not in formally recognised roles. KMs are also not regulated yet they had a very different experience. They described a workplace that provided regular supervision and opportunities for peer support, in addition to access to programmes that fulfilled self-determined professional development needs. They felt valued for their cultural and social determinant expertise, and that they were contributing beyond the service to people and their communities.

“Actually, we were always treated like professionals of our field” – KM, Māori.

“even smaller ‘aha’ moment such as they are smiling, they brought their daughter or someone with them to learn some more, sometimes a community of people sitting there waiting for you” – KM, Māori

Again, this was not discussed in the dietitians focus group.

## Discussion

This study aimed to understand the experiences of the KM, the workforce for Mana Tū, compared with other community-based, non-clinical health workers, and how they support Māori and Pacific Peoples living with T2DM to achieve better outcomes. The analysis of focus group meetings identified similarities and differences between the perspectives of dietitians, CHWs and KM. All three groups agreed that their roles required good relationships with the people they were working with, and an understanding of the contexts in which Māori and Pacific Peoples with T2DM lived. Further, there was an expectation that services were culturally safe (mana enhancing) and required more Māori and Pacific workers and resources. However, CHWs and KM reported a preference for working as part of an MDT and described their experiences as unregulated health workers.

All participants placed high value on whakawhanaungatanga, which for them meant making of culturally meaningful connections with others. They felt this aligned with Māori and Pacific worldviews and cultural values in terms of engaging the collective rather than just individuals. Their perspectives strongly indicated that culturally appropriate communication and relationship building, such as whakawhanaungatanga, encouraged individuals and their whānau to be more open, form relationships and feel safe to discuss health and lives. The concept of whakawhanaungatanga, despite being taught in health curricula continues to be challenged as to how this is integrated into health practice by Māori and non-Māori [19, 21], and should not be regarded as a one-off event to connect with the patient and whānau but, rather, something ongoing that is built upon whilst retaining safe boundaries [22].

In response to the unfair burden of health inequalities disproportionately affecting Māori and Pacific Peoples, there is an urgent need to create culturally safe environments [23]. An Indigenous response to health service delivery is founded on culturally safe practices and concepts [24, 25]. Achieving culturally safe (mana enhancing) practice requires an acknowledgement of one’s own culture, and personal biases in order to understand better the culture of others who are different to oneself, with the intention to further recognise and respect the cultures of people, whānau and communities [26]. In New Zealand, a much-needed evolution from cultural competency to cultural safety is occurring and supports the historical call by Māori seeking a shift in power from traditionally being held by health professionals to shared-care, which is strongly based on their health status and rights with respect to the Treaty of Waitangi [21, 27]. The enablement of culturally responsive healthcare organisations and workforce development [28] is a principal strategy towards the goal of improved cultural competence in health services and systems that better meet the needs of Māori and Pacific Peoples [29]. There are strong calls for this not only to permeate health organisations, but to be sector-wide, and to include social services, education, justice, and research, using systematic and sustainable approaches [25]. An accountable mechanism for providing culturally safe care, as defined by patients and their communities, measured through progress towards achieving health equity [26] is something that may be addressed by the recently announced health reforms. These reforms will establish a Māori Health Authority, with the express aim of enhancing Māori rangatiratanga (governance) for Māori over hauora (health) with greater influence ensured throughout the entire health system [27].

The non-regulated workforce is a priority area needing people whose knowledge and skills are required to respond to the culturally responsive models of care needed to meet the health demands of Māori and Pacific and support to better health outcomes [9]. ‘Kaiāwhina’ is the over-arching term used to describe non-regulated roles in the health and disability sector such as CHWs and KM with essential qualities of resilience, diversity, skill and commitment to support hauora (health) outcomes for all in Aotearoa New Zealand (50% being Māori) [30].

It is important to note that being non-clinical or non-regulated does imply a lack of knowledge or professionalism, as these roles bring the crucial cultural, local and interpersonal skills and expertise that are integral to building relationships and trust with the whānau and communities they work within. A study by Boulton et al. [31] illustrated the restricted career advancement opportunities for Māori CHWs due to the lack of formal role recognition and limited training options. In contrast, the KM described the importance of feeling valued in their roles when they were members of the MDT, received professional development opportunities and had regular peer review. Research suggests that when physicians and non-physician professionals cohesively work together, improved patient outcomes, including better clinical outcome measures and higher patient satisfaction, occur [32]. Considering the recent health reforms in NZ, including the development of a health charter and its focus on a relevant health workforce for communities [29, 33], there is scope to support CHWs and KM in the ways described here.

## Conclusion

This study into three distinct roles for health staff who work with Māori and Pacific with T2DM is a first of its kind, with unique findings resulting. There was deliberate approach to undertake research that was strength-based, with a focus on the system rather than individual people and their behaviour. Importantly, as much as this was about the workers, the ultimate objective was on achieving excellent outcomes for Māori and Pacific Peoples. However, there are several limitations to this study. This is a small project with three focus groups in one area of NZ, and therefore may not apply to other settings or workforce groups. However, it does provide rich insights into the experiences of those people working with an important cohort and has the potential to inform workforce development strategies both here in NZ and for other Indigenous communities.

Indigenous comprehensive health and social programmes that achieve better diabetes outcomes for Māori and Pacific communities require the right workforce. Our study suggests that support for community-based, non-clinical workers to build meaningful and culturally safe relationships with communities, and contribute as valued team members, will improve diabetes outcomes.

## Data Availability

All ethics and research guidelines used for this article are publicly available. This can be accessed on the AUT University research ethics guidelines link: Research Ethics - AUT. The datasets generated and/or analysed during the current study are not publicly available due to Indigenous data sovereignty which protects this data and permission to share transcripts was not sought. Please contact corresponding author: Tania.Mullane@whitiriea.ac.nz for communications regarding accession of data.
